# A *Fusarium verticillioides MAT1-2* Strain near Isogenic to the Sequenced FGSC7600 Strain for Producing Homozygous Multigene Mutants

**DOI:** 10.3390/jof10080592

**Published:** 2024-08-21

**Authors:** Scott E. Gold, Daren W. Brown, Felicia N. Williams, Brian D. Nadon, Vivian T. Vo, Christine E. Miller

**Affiliations:** 1 Toxicology & Mycotoxin Research Unit, Agricultural Research Service, United States Department of Agriculture, US National Poultry Research Center, Athens, GA 30605, USA; 2 Mycotoxin Prevention and Applied Microbiology Research Unit, Agricultural Research Service, United States Department of Agriculture, 1815 N. University Street, Peoria, IL 61604, USA; daren.brown@usda.gov; 3 Genomics and Bioinformatics Research Unit, Agricultural Research Service, United States Department of Agriculture, Athens, GA 30605, USA

**Keywords:** *Fusarium verticillioides*, fumonisins, recombination, single nucleotide polymorphisms, chicken feed

## Abstract

Fungal genetic systems ideally combine molecular tools for genome manipulation and a sexual reproduction system to create an informative assortment of combinations of genomic modifications. When employing the sexual cycle to generate multi-mutants, the background genotype variations in the parents may result in progeny phenotypic variation obscuring the effects of combined mutations. Here, to mitigate this variation in *Fusarium verticillioides*, we generated a *MAT1-2* strain that was near isogenic to the sequenced wild-type *MAT1-1* strain, FGSC7600. This was accomplished by crossing FGSC7600 with the divergent wild-type *MAT1-2* strain FGSC7603 followed by six sequential backcrosses (e.g., six generations) of *MAT1-2* progeny to FGSC7600. We sequenced each generation and mapped recombination events. The parental cross involved twenty-six crossovers on nine of the eleven chromosomes. The dispensable chromosome 12, found in FGSC7603 but lacking in FGSC7600, was not present in the progeny post generation five. Inheritance of complete chromosomes without crossover was frequently observed. A deletion of approximately 140 kilobases, containing 54 predicted genes on chromosome 4, occurred in generation 4 and was retained in generation 5 indicating that these genes are dispensable for growth and both asexual and sexual reproduction. The final *MAT1-2* strain TMRU10/35 is about 93% identical to FGSC7600. TMRU10/35 is available from the Fungal Genetics Stock Center as FGSC27326 and from the ARS Culture Collection as NRRL64809.

## 1. Introduction

Our laboratory studies the biology of mycotoxigenic fungi that are important in food and feed, with a particular focus on the fumonisin producer (reviewed in Marasas, [1]) and frequent corn pathogen, *Fusarium verticillioides* (Saccardo) Nirenberg. Fumonisins are extremely common mycotoxins found in U.S. corn indicating that *F. verticillioides* produces widespread symptomless infections that contaminate the grain. Indeed, of 326 South Eastern U.S. corn samples recently analyzed, Pokoo-Aikins et al. (submitted) found that 100% were contaminated with fumonisins [2]. Fumonisins are associated with severe animal diseases such as equine leukoencephalomalacia causing liquification of the horse brain [3] and porcine pulmonary edema [4] and in human cancers and developmental defects [5]. For this reason, understanding the genetic controls of its colonization of and virulence on maize and the determinants of mycotoxin biosynthesis are of critical need. Filamentous Ascomycota, like *F. verticillioides*, provide a favorable system for genetic analysis for a number of reasons. Prime among these is their haploid nature, allowing unique mutations to be phenotypically expressed. A caveat to this is the fact that many genes are in paralogous families in fungi and thus a full understanding of gene function may require multigene mutants. Multigene mutants can also be informative for determining epistatic genetic relationships. In a previous study [6], we examined the phenotypic effects of single and double mutations in a pair of catalase–peroxidase paralogous genes, FVEG_10866 and FVEG_12888. Although in that study, as expected, the double mutants exhibited a more dramatic impact than those with single mutations, there was an intensity range of phenotypes displayed by the double-mutant progeny. The crosses used to generate the above double-mutant progeny involved two disparately isolated wild-type strains of opposite mating type, FGSC7600 which is *MAT1-1* [7,8] and FGSC7603 which is *MAT1-2* [9,10]. We interpreted the variation in progeny phenotypes to likely be due, in a large part, to allelic variation inherent in the different parent backgrounds (i.e., genetic noise). This led us to the work described here in which we aimed to construct a *MAT1-2* strain that was near isogenic to the standard sequenced wild-type *MAT1-1* strain (FGSC7600) by repeated backcrosses with the *MAT1-1* strain as the recurrent parent. The final strain, the product of seven generations, in theory should have had a recurrent parental allelic specificity of >99%. Here, we identified the major cross-over events through the generations by genome sequencing (Illumina, San Diego, CA, USA) and Single Nucleotide Polymorphisms (SNP) mapping. Our results suggest that our *MAT1-2* final product “near-isogenic” strain, TMRU10/35, shows a 90.9 to 93.5% FGSC7600 allelic specificity. Strain TMRU10/35, stored at the US National Poultry Research Center, has also been deposited with the Fungal Genetic Stock Center as FGSC27326 and at the ARS Culture Collection as NRRL64809 and is available upon request.

## 2. Materials and Methods

### 2.1. Fungal Strains, Growth Conditions, and Media

Carrot agar plates were made as in [11] with slight modifications from 400 g fresh “organic” carrots that were washed, tops trimmed, and diced into cubes of similar sizes. The carrots were added to a 2 L flask with the addition of 400 mL distilled water and autoclaved for 30 min. After cooling, the carrot and water mixture was pureed in batches to create a uniform consistency. The pureed mixture was brought to a total volume of 1000 mL with distilled water and blended again. Then, 250 mL of puree was set aside to be prepared for carrot agar plates; the extra carrot puree was aliquoted in 250 mL volumes into separate freezer bags and stored at −20 °C for future use. To prepare media for plates, 3.5 g of agar (Neogen, Lansing, MI, USA) was mixed with 250 mL carrot puree and then autoclaved once again for 20 min. The medium was cooled to 50 °C in a water bath, then was dispensed into 100 × 15 mm plastic Petri dishes. 

Mating plates were prepared for the crosses by initial inoculation on carrot plates of the *MAT1-2* strain FGSC7603 (NRRL20984; FRCM3703; FJLA00999) for perithecia with minor modification from Klittich and Leslie (1988) [12]. The wild-type recurrent *MAT1-1* strain, FGSC7600 (NRRL20956; FRCM3125; FJLA00149), was inoculated on potato dextrose agar (PDA; Neogen) plates for the production of spermatia. The strains were both inoculated separately at the same time and incubated at 27 °C in the dark [6]. After seven days, the female parent, FGSC7603, was ready for fertilization with the male parent, FGSC7600. Conidia (spermatia) of FGSC7600 were harvested by flooding the PDA plate with 10 mL of sterile water containing 0.1% of Tween 20 and rubbing with a sterile plastic “hockey stick” to dislodge the spores. Female *MAT1-2* plate cultures were male fertilized by spreading 1–2 mL of the *MAT1-1* conidial suspension over the surface. 

For each generation, two independent *MAT1-2* progeny were mated as females to FGSC7600 to produce the subsequent backcross progeny. The more fertile of the two crosses at each generation was used to isolate the progeny for subsequent crosses. In the initial three generations ascospores were isolated from cirrhi as previously described [6] and dilution plated to generate isolated germlings. In subsequent generations ascospores were recovered from the Petri plate lids to which they adhered post ejection followed by dilution plating to generate isolated germlings. This process was repeated to generate seven generations of progeny through sexual crosses.

### 2.2. Genome Sequencing

Whole-genome sequences were generated using a MiSeq (Illumina, Inc.) sequencing platform. DNA was extracted from fungal mycelia grown in GYP medium (2% glucose, 1% peptone, and 0.3% yeast extract) with shaking at 200 rpm at 28 °C for 3 days. The mycelia were harvested by filtration, lyophilized, and ground to a powder. Genomic DNA was then extracted using the Qiagen GenomicTip 20/G protocol (Qiagen, Aarhus, Denmark). DNA sequencing libraries were prepared using the Nextera XT DNA library Preparation Kit (Illumina, Inc.). Sequence reads were imported into CLC Genomics Workbench versions 11.0 (Qiagen), and then screened against genome sequences of 84 bacterial species to remove contaminating DNA from the reagents. The reads were then trimmed to remove low-quality data. The raw sequence data are available from NCBI BioProject PRJNA1133776 and includes the Sequence Read Archive (SRA) accessions SAMN42386886 (strain F1gen1), SAMN42386887 (strain BC1gen2), SAMN42386888 (strain BC2gen3), SAMN42386889 (strain BC3gen4), SAMN42386890 (strain BC4gen5), SAMN42386891 (strain BC5gen6), and SAMN42386892 (strain BC6gen7).

### 2.3. Methods for Fusarium verticillioides Variant Calling

The reference sequence and annotation for parent strain *F. verticillioides* FGSC7600 were downloaded from FungiDB [13], and were indexed for mapping with BWA-MEM (Burrow-Wheeler Aligner (v 0.7.12)) alignment algorithm. Illumina short reads for the recurrent FGSC7600 parent, the non-recurrent FGSC7603 recurrent parent, and the F1 and 6 generations of backcross progeny lines were obtained from BWA-MEM (v 0.7.12) and used to align each Illumina read set to the FGSC7600 reference with standard parameters. BCFtools (v1.9) mpileup was used to calculate read coverage, and bcftools was used to call variants using the multi-allelic model. After calling, the variants were filtered using vcftools (v0.1.15) to keep only biallelic variants, in order to account for errors in the reference sequence and mutations causing spurious multiallelic calls. 

**Figure 1:**
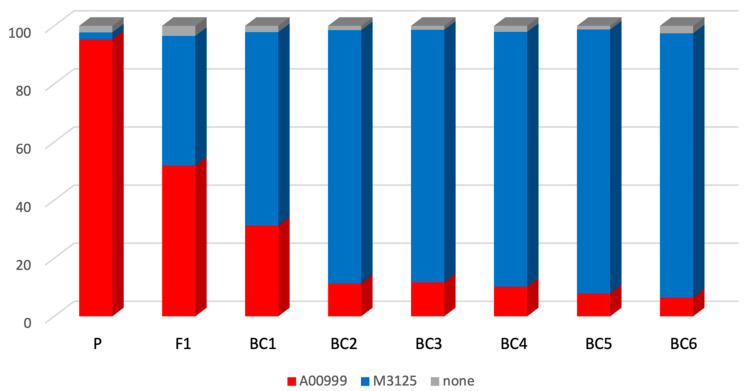
Proportion of parental SNPs per generation. Red represents the nonrecurrent parent FGSC7603 and blue represents the recurrent parent FGSC7600. The final product (strain TMRU10/35; BC6) is approximately 93% identical to the FGSC7600 genotype.

**Figure 2:**
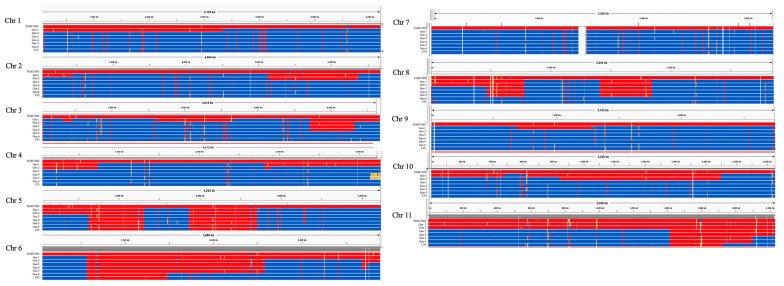
Mapping crossovers through the generations and nonrecurrent parent retention across the eleven *F. verticillioides* chromosomes. Red represents the nonrecurrent parent FGSC7603 and blue represents the recurrent parent FGSC7600 in each generation (Gen#). Generation 1 (Gen1) is the F1 of FGSC7603 and FGSC7600. Generations 2-6 (Gen2-6) are successive backcross progeny. C35 is the 6th backcross and the final seventh generation progeny deposited as FGSC27326 and NRRL64809.

Variants were visualized in IGV v 2.9.4, (Figure 1 and Figure 2) with the non-recurrent FGSC7603 parent depicted in red while the recurrent parent FGSC7600 regions were shown in blue. Yellow regions were absent from the sequence. Loci on chromosomes 5, 6, and 11 were identified that retained “alt” (i.e., non-recurrent FGSC7603 parent) allele calls in the final product. Genes within the boundaries of these loci were identified in the FGSC7600 FungiDB annotation using a custom Python script.

## 3. Results

### 3.1. Production of Progeny

**Figure 3:**
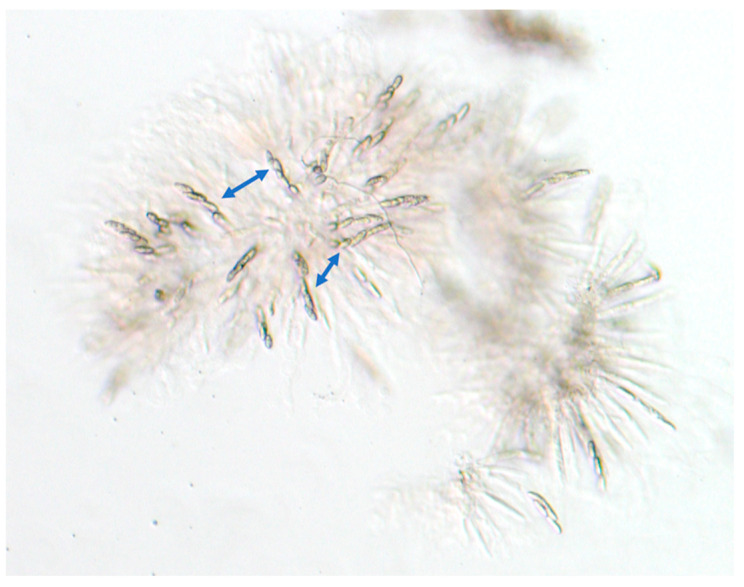
Production of 4-spored asci (blue arrows) by near-isogenic strain TMRU10/35 indicates retention of the *Sk^K^* allele at the *SKC1* locus from the non-recurrent parent FGSC7603.

An initial parental cross was carried out on carrot agar between two wild-type *Fusarium verticillioides* strains of opposite mating type: FGSC7600, *MAT1-1* and FGSC7603, *MAT1-2*. The progeny were single ascospore isolates and *MAT1-2* individuals identified by PCR and observed to have relatively suppressed aerial hyphae to allow easy access to cirrhi for ascospore collection. In the third backcross (BC), and later, the adhered ascospores were collected, post ejection, from the Petri dish lid. Two independently isolated *MAT1-2* progeny were crossed to FGSC7600 for each subsequent generation and the cross that appeared to be more fertile was used to isolate next generation progeny. In total, seven crosses were carried out with the recurrent parent, FGSC7600. The isogenicity of the final (BC6) product was expected to be >99%, not accounting for retention of the FGSC7603 sequences at the *MAT* locus on chromosome 6, and the spore killer (*SKC1*) locus on chromosome 5. The *SKC1* locus has two potential alleles, *Sk^K^* (*Spore killer*) and *Sk^S^* (*Spore killer*-ceptible) [14]. Consistent with the retention of the *Sk^K^* allele derived from FGSC7603 that contains the *SKC1* gene required for spore killing, the progeny strains possessed only four surviving ascospores per ascus rather than the normal eight ascospores [14] (Figure 3). We also unexpectedly observed the retention of sections of chromosome 11 potentially due to the selection of recumbent hyphae, which allowed for better ascospore isolation. The final *MAT1-2* product strain TMRU10/35, was estimated to be approximately 90.9 to 93.5% identical to the FGSC7600 genotype.

### 3.2. Genome Mapping through the Generations

Single nucleotide polymorphisms (SNPs) were mapped and estimates were made for the parental content in each generation (Table 1 and Figure 1). The total genomic SNP count between the parental strains was 243,169, with each of the 11 chromosomes having approximately 20,000 identified SNPs (Table 2). The crossover events were mapped (Figure 2). The contribution of the FGSC7600 genome to the progeny of each successive generation generally followed, at least initially, the expected pattern of an increase in each generation. The F1 through BC2 products demonstrated close alignment to expected ratio of DNA content from each parent; however, later generations disagreed with the predictions dramatically, falling well short of the expected homozygosity in the BC6 product of the seventh cross. 

Interestingly, crossovers were observed (Table 2) in the first generation for all chromosomes except chromosome 7 which was inherited intact from FGSC7600, and also chromosome 11 which was inherited intact from FGSC7603 and did not undergo a crossover event for the first two generations. There was a total of twenty-six crossover events across nine of the eleven chromosomes in the parental cross generating the analyzed F1 strain.

### 3.3. Genotype of BC6 near Isogenic MAT1-2 Strain TMRU10/35

The final near isogenic product of the seventh and final cross (BC6), strain TMRU10/35 (FGSC27326; NRRL64809), retained large sections of chromosomes 5 (35%), 6 (23%) and 11 (15%) from the parental strain FGSC7603, with chromosome 5 retaining two distinct FGSC7603 inherited sections. The coordinates of FGSC7603-inherited segments are as follows: Chr 5 (CM000582.1) 631497-1315560 and 1873373-2702564; Chr 6 (CM000583.1) 568724-1474775; Chr 11 (CM000588.1) 1409238-1717999. The dispensable chromosome 12 found in some strains of *F. verticillioides* is present in FGSC7603 but absent from FGSC7600. Portions of Chr 12 were detected during generation 5 but absent thereafter. Thus, the final product strain, like FGSC7600, lacks Chr 12 and it will not be further discussed.

The mating-type protein MAT1 (FVEG_02491) is encoded by the negative strand located on chromosome 6 [14]. FVEG_02491 in FGSC7600 is located at position (CM000583.1) 957491-958685 which is roughly in the center of the retained FGSC7603 chromosome 6 region, consistent with its *MAT1-2* phenotype. The meiotic drive element protein SKC1 is encoded by the positive strand located on chromosome 5 between FVEG_03164 and FVEG_03165 [15]. In FGSC7600, FVEG_03164 and FVEG_03165 flank position (CM000582.1) 763363 and 764120 on Chr 5, which is well within the first segment of Chr 5 in BC6 which was inherited from FGSC7603.

### 3.4. Increase in Fertility of Final Product

**Figure 4:**
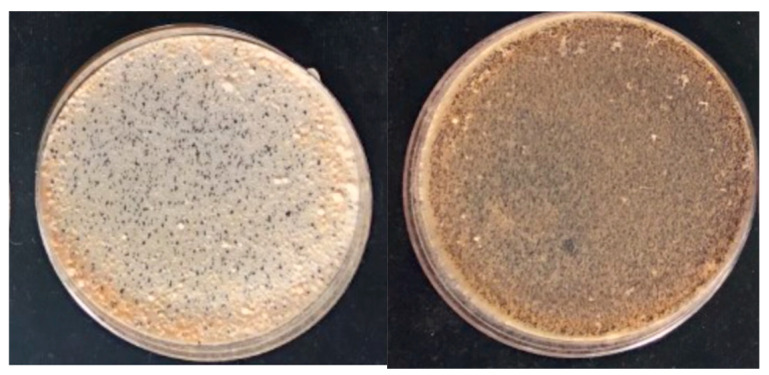
Increased perithecium production observed in crosses with near isogenic strains. **Left**, parental cross FGSC7603 x FGSC7600; and **right** TMRU10/35 (BC6) x FGSC7600. Note the approximately ten-fold more black perithecia on the right compared to the left.

Cross fertility increased in the post parental crosses. The final product, *MAT1-2* strain TMRU10/35 (BC6), is a highly fertile female, when crossed with FGSC7600 as male, generating abundant perithecia and ascospores as compared to the original weak mating reactions with FGSC7603 (Figure 4).

### 3.5. Nonessential Genes at Right Arm of Chromosome 4

**Figure 5:**
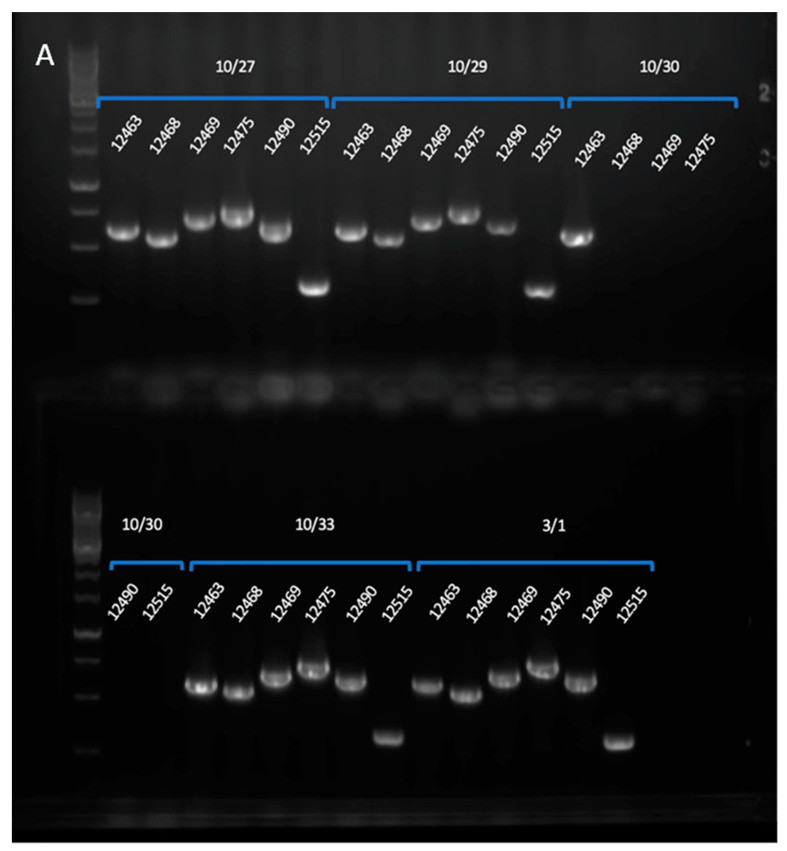

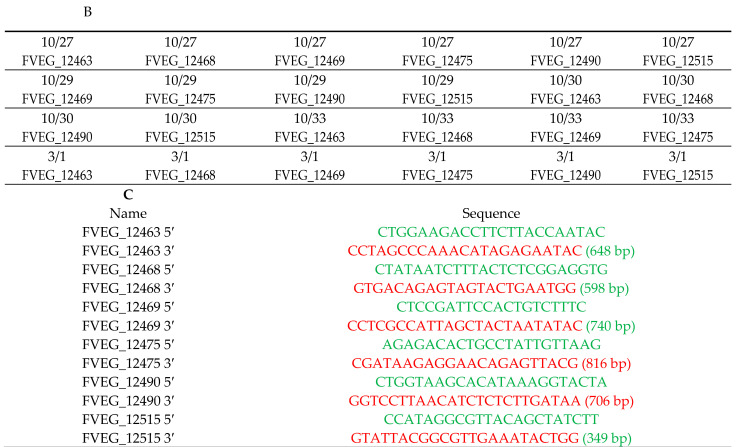
PCR confirmation of deletion of the distal arm of chromosome 4 in generation 5 (BC4). (**A**), agarose gel of amplicons, with GeneRuler 1 kb DNA Ladder (Thermo Fisher Scientific, catalog #SM0311); (**B**), scheme of TMRU strains and genes detected; (**C**), gene specific primer pairs designed and used for this study, green oligonucleotides are forward primers and red are reverse primers, (predicted amplicon length). Strains are TMRU10/27 BC2, TMRU10/29 BC3, TMRU10/30 BC4, TMRU10/33 BC5, TMRU3/1 WT FGSC7600.

In generation 4 and 5, chromosome 4 presents a deletion of approximately 140 kilobase pairs on its distal right arm. FVEG_12469 is the first affected of 54 genes. The deletion of this region was confirmed by PCR using ORF primers designed for this study, and their predicted amplicon lengths are indicated in Figure 5C for four genes, the most proximal deleted gene FVEG_12469, two centrally located genes, FVEG_12475 and FVEG_12490, and the final distal gene FVEG_12515 (Figure 5). As a positive control for the PCR reactions, gene FVEG_12463, which was outside the deleted region, was used to generate a clear product amplicon from the BC4 (Gen5) template.

## 4. Discussion

Here, we produced a strain near isogenic to, and sexually compatible with, the standard sequenced reference strain of *Fusarium verticillioides*. This *MAT1-2* strain is useful for generating multiple gene mutants with minimal genomic variation and consequent phenotypic noise. We carried out a seven-generation sexual backcross recombination approach to generate a near-isogenic compatible mating partner for the commonly used and reference genome strain FGSC7600 (also known as FRCM3125 or F237) [7,8]. The initial cross generated massive chromosomal recombination that was highly subdued in the subsequent backcrosses. The F1 sequenced strain showed 26 observable crossover events involving all but two of the eleven chromosomes. It is clear from the SNP analysis that chromosomes are not infrequently inherited intact from one or the other parent. This is what occurred for chromosome 7 and 11 in generation 1, and also occurred many times for recombinant chromosomes, such as for chromosome 4 in generations 4 through 7. A distal deletion of 140 kilobases pairs on the right arm of chromosome 4 occurred in generation 4, but it returned to the wild-type condition in generation 6. We verified the presence or absence of genes within and outside the putatively deleted region using PCR.

The final product, strain TMRU10/35, shows a 90.9 to 93.5% genome-wide SNP allelic specificity to FGSC7600. This is not as high as expected (>99%) but it represents a dramatic improvement that we expect to significantly reduce progeny phenotypic variation due to allelic variation in the background genome. For example, in previous crosses to generate double mutant progeny, the products theoretically retained an average of 25% FGSC7603 SNP specificity. Envisioning another cross to generate the double mutant as reported in Gao, et al. (2018) [6], the expected progeny would average 5% percent in the FGSC7603 alleles. In the worst-case scenario, FGSC7603 specificity could not exceed the 7–9% remaining in strain TMRU10/35. This suggests a reduction in genetic noise interference of about 5-fold.

Meiotic drive describes a process by which a gene or genes are inherited in most, if not all, progeny, unlike standard genes which are inherited at a rate of 50%. This biased transmission in fungi is frequently achieved through spore killing where typically half of the spores in an ascus die [16]. In *F. verticillioides*, isolates have either a spore-killer (*Sk^K^*) or spore-sensitive (*Sk^S^*) genotype [8]. Crosses between *Sk^K^* isolates or between *Sk^S^* isolates yield asci with eight ascospores while crosses between *Sk^K^* (i.e., FGSC7603) and *Sk^S^* (i.e., FGSC7600) isolates yield asci with four ascospores [14,17]. The gene responsible for *Sk^K^*-mediated spore killing is *SKC1* and is inherited in the four ascospores [15]. Each backcross in this experiment resulted in asci with four ascospores (see Figure 3) indicating that *SKC1* was functional and inherited in the progeny. Based on this, it is not surprising that *SKC1* and a significant segment of Chr 5 flanking *SKC1* from the parent FGSC7603 were retained in the final product strain (CM000582.1 631497–1315560). The presence of an even larger segment of a sequence from Chr 5 (1873373–2702564) as well as a segment from Chr 11 (CM000588.1 1409238–1717999) in the final product strain raise the intriguing possibility that *F. verticillioides* strain FGSC7603 contains two other meiotic drive elements or that *SKC1* requires additional genetic elements to function. Alternatively, these regions of low recombination could be due to the presence of a centromere or other chromosome structural elements [18,19]. However, as noted above, the retention of FGSC7603 sequences on chromosome 11 may be due to selection of a progeny with improved mating efficiency, and/or a flat growth phenotype. FGSC7600 produces extensive aerial hyphae on PDA medium while FGSC7603 possesses a more flattened phenotype making the collection of ascospores easier. 

## 5. Conclusions

Here, we generated a near-isogenic line of the opposite mating type to the standard sequenced *MAT1-1 Fusarium verticillioides* strain FGSC7600 (M3125). This strain is used for the production of double and higher-order mutants through sexual crosses with mutants generated in FGSC7600. Using the final *MAT1-2* product strain resulting from backcross 6 (TMRU10/35, FGSC27326, NRRL64809) provides a predicted 5-fold reduction in allelic variation, likely providing commensurate phenotypic uniformity in generated double mutant progeny. The final *MAT1-2* strain that was near isogenic to our *MAT1-1* laboratory work horse strain FGSC7600 is available as FGSC27326 at the Fungal Genetic Stock Center and as NRRL64809 at the ARS Culture Collection in Peoria, IL. Additionally, all generations of the sequenced progeny leading to NRRL64809 are available at the ARS Culture Collection as NRRL64803 to NRRL64808.

## Figures and Tables

**Table 1:** Expected and actual FGSC7603 non-recurrent parental contributions.

Cross	Male Recurrent Parent	Female Parent	Expected FGSC7603 Genotype of Female	Actual Observed % ^@^	NRRL Product Stock Strain Designation
1 (parental)	FGSC7600 (TMRU3/1, NRRL 20956)	FGSC7603 (TMRU3/8, NRRL 20984)	100%	98%	
2	FGSC7600	F1 (10/24) Gen1	50%	54%	64803
3	FGSC7600	BC1 (10/25) Gen2	25%	32%	64804
4	FGSC7600	BC2 (10/27) Gen3	12.5%	12%	64805
5	FGSC7600	BC3 * (10/29) Gen4	6.25%	12%	64806
6	FGSC7600	BC4 (10/30) Gen5	3.12%	10%	64807
7	FGSC7600	BC5 (10/33) Gen6	1.56%	8%	64808
Product	BC6 (TMRU10/35; FGSC27326) Gen7		0.78%	7%	64809

^@^ calculated as follows FGSC7603/(FGSC7600 + FGSC7603). Rounded to nearest whole number. * Backcross 3 (BC3) SNP analysis indicated that a deletion occurred at the distal right arm of chromosome 4 but that otherwise it was identical to the sequenced BC2 strain. This deletion was retained in the BC4 strain and the sequence within this region was recovered to the wild-type condition in the BC5 strain.

**Table 2:** Observed crossover events for each chromosome.

Chromosome	SNPs	Generation:Crossover(s)	Maximum % FGSC7600 Generation
1	25,874	1:1, 2:1 *	100% generation 2
2	21,797	1:3, 2:1, 3:2 *	100% generation 3
3	19,070	1:5, 5:2 *	100% generation 5
4	28,516	1:2, 2:1, 3:3 *	100% generation 3
5	22,138	1:5, 6:2	65% generation 6
6	23,264	1:1, 2:2, 3:1, 5:1, 6:1	77% generation 6
7	18,434	1:0 *	100% generation 1
8	26,083	1:3, 3:1, 6:4 *	100% generation 6
9	17,656	1:2, 2:2 *	100% generation 2
10	20,639	1:3, 2:1, 3:2 *	100% generation 3
11	19,698	3:1, 5:1, 7:1	85% generation 7

* In the generation producing 100% FGSC7600 chromosome identity, homozygosity is often likely due to inheritance of the intact FGSC7600 recurring parent chromosome, as observed in generation 1 for chromosome 7. A similar situation occurred in the first 2 generations for chromosome 11, but in that case, inheritance was of the full non-recurrent parent chromosome.

## Data Availability

The data presented in this study are available within this article and the sequence data described is available at NCBI BioProject PRJNA1133776. The seven generation strains (Gen1–Gen7) are deposited at the Agricultural Research System (ARS) Culture Collection (https://nrrl.ncaur.usda.gov). The final *Mat1-1* near isogenic line product (Gen7) strain is deposited at the Fungal Genetics Stock Center as FGSC27326.
